# Baseline erythrocyte sedimentation rate is associated with mortality in dogs with chronic kidney disease

**DOI:** 10.3389/fvets.2026.1924110

**Published:** 2026-10-22

**Authors:** Chang-Hun Moon, Jae-Beom Joo, Woong-Bin Ro, Chang-Min Lee

**Affiliations:** 1 Department of Veterinary Internal Medicine, College of Veterinary Medicine and BK21 FOUR program, Chonnam National University, Gwangju, Republic of Korea; 2 SNC Animal Medical Center, Seoul, Republic of Korea; 3 Shine Animal Medical Center, Seoul, Republic of Korea

**Keywords:** inflammatory biomarker, multi-biomarker risk score, prognosis, risk stratification, survival analysis

## Abstract

**Background:**

Chronic kidney disease (CKD) in dogs has variable survival outcomes and systemic complications not fully captured by renal excretory markers. Erythrocyte sedimentation rate (ESR) may reflect cumulative systemic disease burden, but its prognostic relevance in canine CKD remains unclear.

**Objective:**

To evaluate the prognostic value of baseline ESR in dogs with CKD and its contribution to mortality risk stratification with conventional clinical and renal variables. Animals: A total of 272 dogs were included: 145 healthy controls and 127 dogs with CKD.

**Methods:**

Associations among ESR, clinicopathological variables, CKD stage, and survival were assessed. Prognostic factors were evaluated using receiver operating characteristic (ROC) curve analysis and Cox proportional hazards regression. Two exploratory composite risk scores were constructed from multivariable Cox regression coefficients: one excluding ESR and one incorporating ESR. Their apparent performance was compared using areas under the ROC curve (ROC-AUCs) and Kaplan–Meier survival analyses.

**Results:**

Non-survivors had a higher ESR than survivors [median (IQR), 21 (13–40) vs. 11 (8–14) mm/h; *p* < 0.0001]. The optimal ESR cut-off was 16 mm/h, and ESR alone had an ROC-AUC of 0.79 [95% confidence interval (CI), 0.71–0.88]. An ESR of ≥16 mm/h remained associated with mortality in multivariable Cox regression analysis (hazard ratio, 3.42; 95% CI, 1.78–6.64). The scores incorporating and excluding ESR had apparent ROC-AUCs of 0.87 (95% CI, 0.81–0.93) and 0.85 (95% CI, 0.79–0.92), respectively, with no statistically significant difference between them (paired DeLong test, *p* = 0.39).

**Conclusion:**

Baseline ESR was associated with disease severity and all-cause mortality in dogs with CKD. Baseline ESR may provide complementary prognostic information when interpreted with conventional renal and clinical variables. The ESR-inclusive composite score should be considered exploratory and requires external validation.

## 1 Introduction

The erythrocyte sedimentation rate (ESR) is a hematological marker that reflects the sedimentation of erythrocytes in anticoagulated whole blood. Its elevation is mainly driven by erythrocyte aggregation, which is promoted by plasma proteins such as fibrinogen and immunoglobulins that reduce the negative surface charge of red blood cells and facilitate rouleaux formation (1, 2). Although ESR is nonspecific, it can reflect the cumulative burden of systemic inflammation and has been investigated as a clinically relevant inflammatory and prognostic marker in dogs (1–4).

Chronic kidney disease (CKD) is a progressive and irreversible disorder characterized by persistent structural or functional abnormalities of the kidney. In dogs, CKD is commonly staged according to the International Renal Interest Society (IRIS) guidelines using serum creatinine and symmetric dimethylarginine (SDMA), followed by substaging based on proteinuria and systemic blood pressure (5). However, CKD is not limited to renal excretory dysfunction. As the disease progresses, dogs may develop systemic complications including uremia, anemia, mineral imbalance, metabolic acidosis, proteinuria, systemic hypertension, nutritional deterioration, and cardiovascular comorbidities (5, 6).

The prognosis of canine CKD is highly variable. Previous studies have reported that survival is associated with multiple factors, including IRIS stage, serum creatinine, blood urea nitrogen (BUN), phosphorus, calcium-phosphorus product, urine protein-to-creatinine ratio (UPC), body condition, muscle condition, and hematological abnormalities (7–10). These findings suggest that a single renal parameter is insufficient to fully represent the clinical severity and mortality risk of CKD. Instead, prognostic assessment should consider the cumulative disease burden created by renal dysfunction, metabolic derangement, inflammation, and comorbid disease.

In this context, ESR may be clinically useful in dogs with CKD. Although ESR is nonspecific and can be affected by various non-renal factors, including anemia and altered plasma protein composition (1), its broad responsiveness to systemic inflammation and chronic disease burden (1–4) may allow it to capture systemic consequences of CKD that are not fully reflected by renal excretory markers alone (5, 6). Therefore, ESR may serve as an integrative biomarker that complements conventional renal markers such as BUN and creatinine, rather than functioning as a standalone disease-specific prognostic marker (5–7).

A multi-biomarker approach may overcome this limitation by integrating variables that represent different pathophysiological aspects of CKD. Renal excretory markers (BUN and creatinine), mineral metabolism parameters (calcium, phosphate, and Ca × P), electrolyte and acid–base variables (potassium and bicarbonate), inflammatory indicators, proteinuria, hypertension, and cardiovascular comorbidities may each contribute different prognostic information. Combining these variables into a weighted risk score may provide a more comprehensive estimate of mortality risk than individual biomarkers alone.

Therefore, the primary aim of this study was to evaluate the clinical and prognostic relevance of baseline ESR in dogs with CKD. We hypothesized that ESR would be associated with CKD severity and all-cause mortality. As a secondary exploratory analysis, we constructed composite risk scores with and without ESR to examine whether ESR could provide additional prognostic information when interpreted together with conventional renal and clinical variables.

## 2 Materials and methods

### 2.1 Population selection

This single-center retrospective cohort study included dogs presented to a referral animal hospital between October 2023 and January 2026, identified through a review of medical records. Written informed consent for the use of clinical data for research purposes was obtained from the owners at admission. Dogs were eligible for inclusion if sufficient clinical and clinicopathological data were available to classify them as either healthy controls or dogs with chronic kidney disease (CKD). The study population was divided into two groups: a healthy control group and a CKD group.

All dogs underwent clinical evaluation, including history taking, physical examination, complete blood count (CBC), serum biochemical analysis, electrolyte analysis, and urinalysis. Thoracic radiography, abdominal ultrasonography, and echocardiography were performed when required for clinical assessment and group classification. Dogs with incomplete baseline data for CKD diagnosis, CKD staging, or erythrocyte sedimentation rate (ESR) measurement were excluded from the relevant analyses.

Survival data were obtained from medical records and follow-up communication. Overall survival was defined as the interval from the date of initial evaluation and ESR measurement to death or the last follow-up. Dogs that were alive at the last follow-up were censored in survival analyses.

The study protocol was approved by the Institutional Animal Care and Use Committee of Chonnam National University. The approval number was CNU-IACUC-2026-41.

### 2.2 Animals

A total of 272 dogs were included in this study. The healthy control group consisted of 145 dogs, and the CKD group consisted of 127 dogs.

Healthy control dogs were presented for routine health screening, elective neutering, dental scaling, or mild transient clinical signs that were not associated with clinically relevant systemic disease. Dogs were classified as clinically healthy only when history, physical examination, CBC, serum biochemical analysis, electrolyte analysis, urinalysis, and available diagnostic imaging identified no clinically relevant abnormalities. Therefore, this group was interpreted as a clinically healthy control population rather than an age-matched disease-free reference population.

Dogs in the CKD group were diagnosed and staged according to the International Renal Interest Society (IRIS) recommendations. For each dog, the following data were collected: breed, age, sex, neutering status, body weight, follow-up duration, survival status, IRIS CKD stage, proteinuria substage, blood pressure substage, ESR, hematological variables, serum biochemical variables, electrolyte variables, urinalysis results, and clinically relevant comorbidities.

### 2.3 Diagnosis, staging, and substaging of chronic kidney disease

Dogs were diagnosed with CKD based on persistent renal functional and/or structural abnormalities. Chronicity was established using previous medical records or repeated clinicopathological testing when available. In dogs without serial data, chronicity was inferred from ultrasonographic findings compatible with chronic renal remodeling, such as cortical thinning, decreased corticomedullary distinction, irregular renal contour, or other structural changes consistent with chronic renal disease, together with the absence of evidence supporting acute kidney injury, pre-renal azotemia, or post-renal disease.

Dogs with CKD were staged according to IRIS CKD staging guidelines using fasting serum creatinine and/or SDMA concentrations measured in clinically stable patients (5). Based on serum creatinine concentration, dogs were classified as stage 1 when creatinine was <1.4 mg/dL, stage 2 when creatinine was 1.4–2.8 mg/dL, stage 3 when creatinine was 2.9–5.0 mg/dL, and stage 4 when creatinine was >5.0 mg/dL. When SDMA and creatinine indicated different stages, both values were interpreted together with hydration status, muscle mass, clinical condition, and repeated measurements when available.

Proteinuria substaging was performed using the urine protein-to-creatinine ratio (UPC). Dogs were classified as non-proteinuric when UPC was <0.2, borderline proteinuric when UPC was 0.2–0.5, and proteinuric when UPC was >0.5. Proteinuria was interpreted after excluding pre-renal and post-renal causes when possible.

Blood pressure substaging was performed using systolic blood pressure. Dogs were classified as having minimal risk of target organ damage when systolic blood pressure was <140 mmHg, low risk when systolic blood pressure was 140–159 mmHg, moderate risk when systolic blood pressure was 160–179 mmHg, and high risk when systolic blood pressure was ≥180 mmHg (5).

For prognostic analyses, proteinuria and systemic hypertension were converted into binary categorical variables. Proteinuria was defined as present when UPC was >0.5 and absent when UPC was ≤0.5. Systemic hypertension was defined as present when systolic blood pressure was ≥160 mmHg and absent when systolic blood pressure was <160 mmHg. These binary variables were coded as 1 when present and 0 when absent.

Dogs were excluded from the CKD group if they had evidence of acute kidney injury, pre-renal azotemia, post-renal azotemia, obstructive urolithiasis, pyelonephritis, active urinary tract infection, severe acute systemic inflammation such as sepsis or acute pancreatitis, malignant neoplasia, or recent administration of nephrotoxic drugs, blood transfusion, or immunosuppressive therapy within 4 weeks before evaluation. These exclusion criteria were applied to minimize potential confounding effects on ESR and systemic inflammatory biomarkers.

### 2.4 Erythrocyte sedimentation rate measurement

The ESR was measured using an automated ESR analyzer, MINI-PET (DIESSE Diagnostica Senese S.p.A., Siena, Italy), according to the manufacturer’s instructions and previously described methods (11). Blood samples were obtained by jugular venipuncture and collected into 1 mL K3-EDTA tubes (IDEXX Laboratories, Westbrook, ME, USA). The same EDTA blood sample used for CBC analysis was used for ESR measurement.

Complete blood count (CBC) analysis was performed using a ProCyte Dx hematology analyzer (IDEXX Laboratories, Westbrook, ME, USA). As part of the routine diagnostic workup at our hospital, ESR measurement was performed within minutes after venipuncture, and no blood samples were stored before ESR analysis. After gentle mixing of the EDTA blood tube, the sample was placed in the MINI-PET analyzer. The device performed an automated optical reading, and ESR results were displayed after 14 min. All ESR values were recorded as mm/h.

### 2.5 Clinical and laboratory variables

Inflammatory and prognostic variables were selected based on their reported associations with systemic inflammation, CKD progression, and survival in dogs with CKD (7–10, 12–15). The selected inflammatory biomarkers included ESR, white blood cell count (WBC), neutrophil-to-lymphocyte ratio (NLR), platelet-to-lymphocyte ratio (PLR), albumin-to-globulin ratio (A/G ratio), and C-reactive protein (CRP).

The selected renal, metabolic, electrolyte, and clinical variables included BUN, serum creatinine, calcium-phosphorus product (Ca × P), potassium, bicarbonate, proteinuria, systemic hypertension, and congestive heart failure associated with myxomatous mitral valve disease (MMVD stage C or higher).

Hematological variables, including WBC, neutrophil count, lymphocyte count, platelet count, and hematocrit, were measured using a ProCyte Dx hematology analyzer. Complete blood count analysis was performed on fresh EDTA-anticoagulated blood samples on the day of collection as part of routine clinical testing. The NLR was calculated by dividing the neutrophil count by the lymphocyte count. The PLR was calculated by dividing the platelet count by the lymphocyte count.

Serum biochemical variables, including BUN, creatinine, albumin, globulin, and CRP, were measured using an automated serum chemistry analyzer (Catalyst Dx, IDEXX Laboratories, Westbrook, ME, USA). Serum biochemical and electrolyte analyses were performed on the day of sample collection according to the hospital’s routine laboratory workflow. Electrolyte and acid–base variables, including potassium and bicarbonate, were measured using the hospital’s routine automated electrolyte or blood gas analyzer.

Mineral variables, including total calcium and phosphate, were measured as part of routine biochemical or electrolyte testing. Serum total calcium concentration was corrected for serum albumin concentration using the following equation: corrected calcium (mg/dL) = measured total calcium (mg/dL) – albumin (g/dL) + 3.5. The calcium-phosphorus product (Ca × P) was calculated by multiplying the total calcium concentration by the phosphate concentration, both expressed in mg/dL, and was reported as mg^2^/dL^2^. The Ca × P was included as a mineral metabolism-related prognostic variable because previous studies have reported its association with outcome in dogs with CKD (9, 10).

Urinalysis was performed using urine samples collected at the time of initial evaluation. The UPC was used to assess proteinuria and to perform IRIS proteinuria substaging. The SBP was used for IRIS blood pressure substaging.

Concurrent congestive heart failure was recorded when dogs had MMVD stage C or higher, based on clinical signs, thoracic radiographic findings, and echocardiographic assessment according to established veterinary cardiology guidelines (15).

### 2.6 Exploratory assessment of ESR contribution using composite risk scores

To evaluate the prognostic contribution of ESR in the context of conventional renal and clinical variables, exploratory composite risk scores were constructed using Cox proportional hazards regression analysis. This analysis was intended to assess the apparent contribution of ESR to risk stratification within this cohort, rather than to establish a fully validated clinical prediction model. Therefore, the composite scores were interpreted as exploratory tools, and their clinical applicability requires independent validation.

Candidate variables were selected from inflammatory, renal, metabolic, electrolyte, and clinical factors considered relevant to CKD prognosis. First, candidate variables were compared between survivors and non-survivors in the CKD group. For continuous variables that differed according to survival status, receiver operating characteristic (ROC) curve analysis was performed to determine cohort-derived cut-off values for all-cause mortality prediction. Cut-off values were determined using the Youden index, which maximizes the sum of sensitivity and specificity.

Predefined categorical clinical variables, including proteinuria, systemic hypertension, and MMVD stage C or higher (defined as described above), were not subjected to ROC-derived cut-off analysis.

After cut-off determination or clinical categorization, candidate prognostic variables were converted into binary variables according to the presence or absence of each risk factor. Each variable was coded as 1 when the risk factor was present and 0 when absent. Univariable Cox proportional hazards regression analysis was performed to evaluate the association between each candidate variable and all-cause mortality. Variables with *p* < 0.05 in univariable analysis were entered into the multivariable Cox proportional hazards regression models. Systemic hypertension (*p* = 0.066) was also entered, regardless of its univariable *p* value, because of its established clinical relevance to CKD progression.

To examine the contribution of ESR, two multivariable Cox regression models were constructed. The first model excluded ESR and was used to construct a multi-biomarker CKD risk score without ESR, defined as the MCB score. The second model incorporated ESR and was used to construct a multi-biomarker CKD risk score incorporating ESR, defined as the MCBE score.

Risk scores were generated using beta coefficients derived from the multivariable Cox proportional hazards regression models. The general equation for the weighted linear predictor was as follows:


Linear predictor=β1X1+β2X2+β3X3+…

where *β* represents the beta coefficient estimated from the multivariable Cox regression model, and X represents the binary coding of each risk factor. Each risk factor was coded as 1 when present and 0 when absent.

The MCB and MCBE scores were evaluated in the same cohort from which they were constructed. Therefore, ROC and Kaplan–Meier analyses of these scores were interpreted as estimates of apparent prognostic performance. The detailed components and assigned weights of the MCB and MCBE scores are presented in the Results section.

### 2.7 Statistical methods

Normality of continuous variables was assessed using the Shapiro–Wilk test. Variables with *p* < 0.05 were considered non-normally distributed. Normally distributed variables were expressed as mean ± standard deviation, whereas non-normally distributed variables were expressed as median and interquartile range. For comparisons between two groups, normally distributed variables were analyzed using the unpaired *t*-test, and non-normally distributed variables were analyzed using the Mann–Whitney *U* test. For comparisons among multiple groups, including healthy control dogs and dogs with different IRIS CKD stages, one-way analysis of variance or the Kruskal–Wallis test was used according to data distribution. *Post hoc* comparisons were performed to identify differences between individual groups.

Categorical variables, including proteinuria, systemic hypertension, and MMVD stage C or higher, were presented as number and percentage. These variables were compared between survivors and non-survivors using Fisher’s exact test. Percentages were calculated within each survival group.

For correlation, multiple linear regression, and Cox proportional hazards regression analyses, binary categorical variables were coded as 1 when the risk factor was present and 0 when absent. Cut-off values were not derived from ROC analysis for predefined categorical clinical variables, including proteinuria, systemic hypertension, and MMVD stage C or higher.

Pearson’s correlation analysis was performed as an exploratory assessment of linear associations between ESR and clinicopathological variables in dogs with CKD. For binary categorical variables, Pearson’s correlation analysis was interpreted as a point-biserial association. Correlation coefficients were interpreted as follows: weak correlation, 0.1–0.3; moderate correlation, 0.4–0.6; and strong correlation, 0.7–0.9 (16).

Multiple linear regression analysis was performed to identify variables associated with ESR levels in dogs with CKD. Variables were selected based on correlation analysis and clinical relevance. Regression estimates and 95% confidence intervals were calculated.

Receiver operating characteristic curve analysis was performed to evaluate the apparent prognostic accuracy of continuous biomarkers and composite risk scores for all-cause mortality prediction. The ROC-AUC, optimal cut-off value, sensitivity, and specificity, with their 95% CIs, were calculated; 95% CIs for sensitivity and specificity were calculated using the Wilson score method. Optimal cut-off values were determined using the Youden index. The ROC-AUCs of the MCB and MCBE scores were compared using the DeLong test for paired ROC curves; because both scores were constructed and evaluated within the same cohort, these ROC-AUCs were regarded as apparent estimates.

Cox proportional hazards regression analysis was used to identify variables associated with all-cause mortality. Continuous variables were dichotomized using ROC-derived cut-off values, whereas predefined clinical variables were entered as binary categorical variables. Hazard ratios, beta coefficients, 95% confidence intervals, and *p* values were calculated. Univariable Cox regression analysis was followed by multivariable Cox regression analysis.

Kaplan–Meier survival analysis was performed to evaluate survival differences according to ESR, MCB score, and MCBE score. Dogs with CKD were stratified into four ordered groups based on ESR values, MCB score, and MCBE score. Median survival time was calculated for each group. Survival curves were compared using the log-rank test. Because the Cox models and composite scores were generated and evaluated within the same cohort, hazard ratios, ROC curves, and Kaplan–Meier stratification were interpreted as cohort-specific estimates of apparent prognostic association rather than externally validated predictive performance.

All statistical tests were two-sided, and a *p* value < 0.05 was considered statistically significant. All statistical analyses were performed using GraphPad Prism version 11.0 (GraphPad Software Inc., San Diego, CA, USA).

## 3 Results

### 3.1 Study population

A total of 272 dogs were included in this study, consisting of 145 healthy control dogs and 127 dogs with chronic kidney disease (CKD). Dogs in the healthy control group had no history of systemic disease and showed no clinically relevant abnormalities based on physical examination, routine clinicopathological testing, and available diagnostic imaging. Dogs in the CKD group were diagnosed and staged according to the International Renal Interest Society (IRIS) guidelines.

The median age was 5.2 years in the healthy control group (IQR, 2.5–9.5 years; range, 0.1–16.1 years) and 12.5 years in the CKD group (IQR, 10.5–15.4 years; range, 4.2–20.0 years). The median body weight was 7.1 kg in the healthy control group (IQR, 5.1–8.5 kg) and 6.4 kg in the CKD group (IQR, 4.7–8.1 kg). Detailed signalment data, including sex and breed distribution, are presented in Table 1.

**Table 1:** Signalments of all dogs included in the study.

Category	Variables	Details
Age		Control (*n* = 145); median 5.2 years (range, 0.1–16.1 years, IQR: [2.5–9.5 years])
		CKD (*n* = 127); median 12.5 years (range:4.2–20 years, IQR: [10.5–15.4 years])
Body weight		Control (*n* = 145); median 7.1 kg, IQR: [5.1–8.5 kg])
		CKD (*n* = 127); median 6.4 kg, IQR: [4.7–8.1 kg])
Sex		Control (*n* = 145); Spayed female (*n* = 57), Intact female (*n* = 10), Castrated male (*n* = 69), Male (*n* = 9) CKD (*n* = 127); Spayed female (*n* = 54), Intact female (*n* = 3), Castrated male (*n* = 56), Male (*n* = 14)
Breeds	Pure breeds (*n* = 230) and mixed breeds (*n* = 42)	Control (*n* = 145): Toy poodle (*n* = 27), mixed (*n* = 23), Bichon Frise (*n* = 19), Maltese (*n* = 17), Pomeranian (*n* = 14), Chihuahua (*n* = 7), Coton de Tuléar (*n* = 6), Yorkshire Terrier (*n* = 5), Spitz (*n* = 4), Welsh Corgis (*n* = 4), Italian greyhound (*n* = 3), Shih Tzu (*n* = 3), Labrador retriever (*n* = 2), Shiba Inu (*n* = 2), American Cocker Spaniel, Beagle, Brussels Griffon, Cane Corso, Doberman pinscher, Golden Retriever, Pekingese, Siberian husky, Miniature pinscher (*n* = 1 each) CKD (*n* = 127): Maltese (*n* = 26), Pomeranian (*n* = 23), Mixed (*n* = 19), Toy poodle (*n* = 14), Chihuahua (*n* = 9), Shetland Sheepdog (*n* = 8), Shih Tzu (*n* = 8), Bichon Frise (*n* = 6), Yorkshire Terrier (*n* = 3), Coton de Tuléar (*n* = 2), Jindo dog (*n* = 2), American cocker spaniel, Boston terrier, French bulldog, Golden retriever, Miniature pinscher, Old English sheepdog, Welsh Corgis (*n* = 1 each)

Data are presented as median, range, and interquartile range for age, median and interquartile range for body weight, and number of dogs for sex and breed distribution. The study population consisted of 145 healthy control dogs and 127 dogs with chronic kidney disease. CKD, chronic kidney disease; IQR, interquartile range.

Among the 127 dogs with CKD, 53 dogs were classified as IRIS stage 1 (41.7%), 46 dogs as stage 2 (36.2%), 14 dogs as stage 3 (11.0%), and 14 dogs as stage 4 (11.0%). Survival status was available for all dogs with CKD. Seventy-five dogs (59.1%) were classified as survivors, and 52 dogs (40.9%) were classified as non-survivors.

### 3.2 Inflammatory biomarker profiles and ESR according to CKD stage

Inflammatory biomarker profiles were compared between healthy control dogs and dogs with CKD. The median ESR was significantly higher in dogs with CKD than in healthy control dogs, with values of 13 mm/h (IQR, 10–18 mm/h) and 9 mm/h (IQR, 3–11 mm/h), respectively (*p* < 0.0001).

Other inflammatory biomarkers also differed between the two groups. The median CRP concentration was significantly higher in dogs with CKD than in healthy control dogs, with values of 2.1 mg/dL and 0.6 mg/dL, respectively (*p* < 0.0001). The median NLR was 5.1 in dogs with CKD and 2.8 in healthy control dogs (*p* < 0.0001). The median PLR was 172.4 in dogs with CKD and 110.3 in healthy control dogs (*p* < 0.0001). The median WBC count was 13.53 × 10^9^/L in dogs with CKD and 10.3 × 10^9^/L in healthy control dogs (*p* < 0.0001). In contrast, the median A/G ratio was lower in dogs with CKD than in healthy control dogs, with values of 0.8 and 0.9, respectively (*p* < 0.001). These results are summarized in Supplementary Table S1.

Age and ESR values according to IRIS CKD stage are summarized in Table 2. The median ages were 5.2 years in healthy control dogs and 11.9, 14.0, 16.0, and 10.8 years in dogs with CKD stages 1, 2, 3, and 4, respectively. The corresponding median ESR values were 9, 13, 12, 15, and 37 mm/h, respectively.

**Table 2:** Descriptive age distribution and ESR comparisons according to IRIS CKD stage and survival status in dogs with CKD.

Variables	Age, median (IQR)	ESR (mm/h), median (IQR)	ESR *p* value	ESR *Post hoc* comparison
CKD IRIS stage (*n*)			vs. healthy control	*p* value
Control (*n* = 145)	5.2 (2.5–9.5)	9 (3–11)	Reference	–
CKD 1 (*n* = 53)	11.9 (8.9–14.9)	13 (7–16)	0.054	–
CKD 2 (*n* = 46)	14 (12.1–15.4)	12 (10–14)	0.059	–
CKD 3 (*n* = 14)	16 (13.7–16.8)	15 (13–24)	<0.0001	CKD 1 (0.013) CKD 2 (0.019)
CKD 4 (*n* = 14)	10.8 (9–11.6)	37 (21–56)	<0.0001	CKD 1 (<0.0001) CKD 2 (<0.0001) CKD 3 (<0.001)
Survival outcome			vs. survivors	
CKD Alive (*n* = 75)	12.2 (9.5–15)	11 (8–14)	Reference	–
CKD Deceased (*n* = 52)	13.5 (11–16)	21 (13–40)	<0.0001	–

Data are presented as median and interquartile range. Age values are provided descriptively and were not statistically compared in this table. All p values and post hoc comparisons shown in this table refer only to ESR comparisons. The ESR values were compared among healthy control dogs and dogs with different IRIS CKD stages, and post hoc pairwise comparisons are shown when statistically significant. These ESR values were also compared between CKD survivors and non-survivors. CKD, chronic kidney disease; ESR, erythrocyte sedimentation rate; IRIS, International Renal Interest Society; IQR, interquartile range.

No significant difference in ESR was observed between healthy control dogs and dogs with CKD stage 1 (*p* = 0.054) or CKD stage 2 (*p* = 0.059). In contrast, ESR was significantly higher in dogs with CKD stage 3 and stage 4 than in healthy control dogs (*p* < 0.0001 for both comparisons). Among dogs with CKD, ESR was significantly higher in stage 3 than in stage 1 (*p* = 0.013) and stage 2 (*p* = 0.019). Dogs with CKD stage 4 had significantly higher ESR values than dogs with stage 1, stage 2, and stage 3 CKD (*p* < 0.0001, *p* < 0.0001, and *p* < 0.001, respectively). These stage-based results are presented in Table 2 and Figure 1.

**Figure 1:**
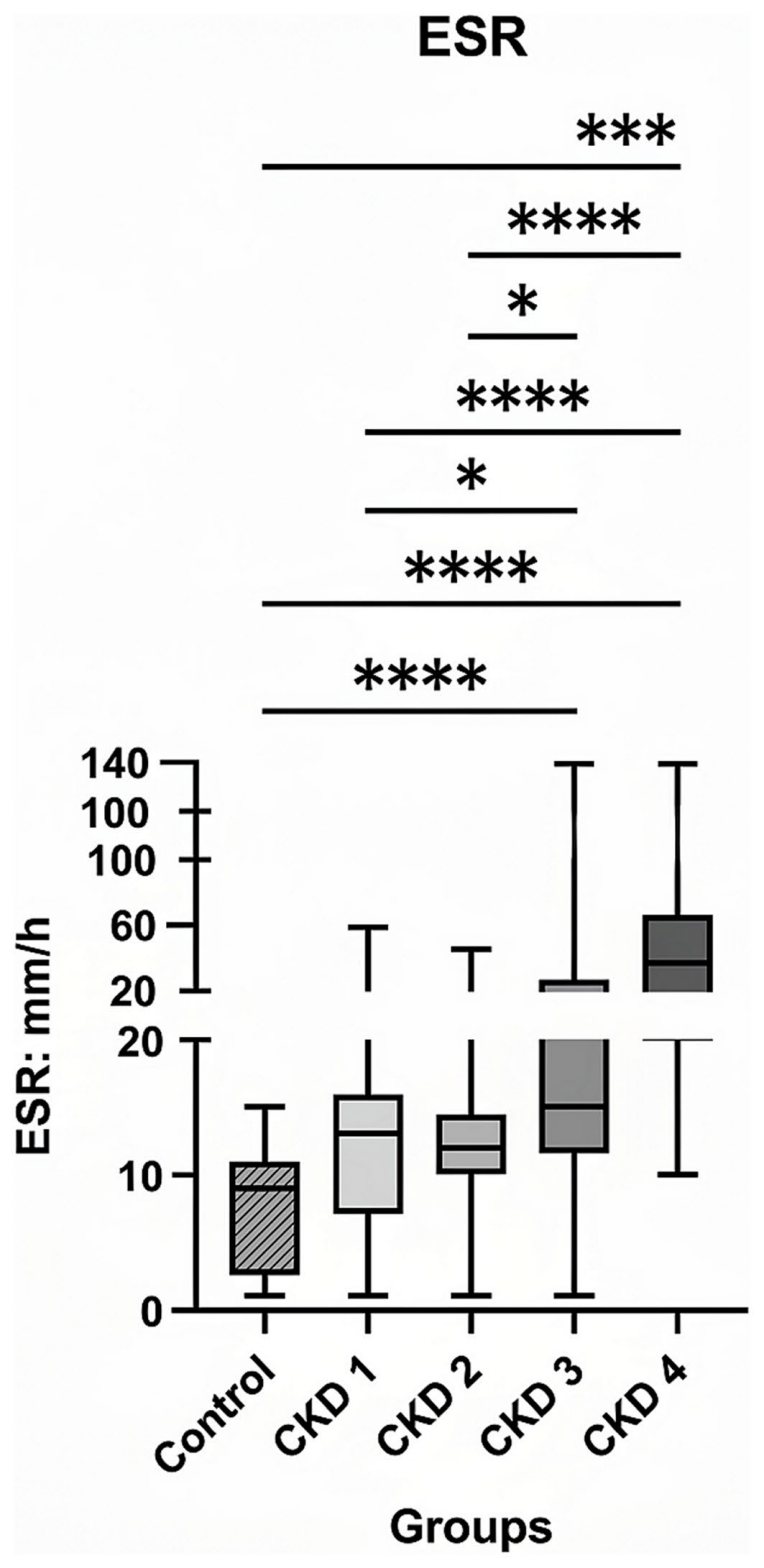
Comparison of ESR levels between healthy control dogs and dogs with different IRIS CKD stages. Box-and-whisker plots show ESR values in healthy control dogs and dogs with IRIS CKD stages 1–4. Boxes represent interquartile ranges, horizontal lines represent medians, and whiskers represent ranges. The median ESR values were 9 mm/h in healthy control dogs, 13 mm/h in CKD stage 1, 12 mm/h in CKD stage 2, 15 mm/h in CKD stage 3, and 37 mm/h in CKD stage 4. Dogs with CKD stage 3 and stage 4 had significantly higher ESR values than healthy control dogs. Among dogs with CKD, stage 3 dogs had significantly higher ESR values than stage 1 and stage 2 dogs, and stage 4 dogs had significantly higher ESR values than stage 1, stage 2, and stage 3 dogs. ESR, erythrocyte sedimentation rate; CKD, chronic kidney disease; IRIS, International Renal Interest Society. Significance levels are indicated as follows: *p < 0.05; ***p < 0.001; ****p < 0.0001. Exact *p* values are presented in Table 2.

According to survival status, the median ages were 12.2 years in survivors and 13.5 years in non-survivors. In contrast, the median ESR was 11 mm/h in survivors and 21 mm/h in non-survivors, and ESR was significantly higher in non-survivors than in survivors (*p* < 0.0001). These results are presented in Table 2 and Figure 2.

**Figure 2:**
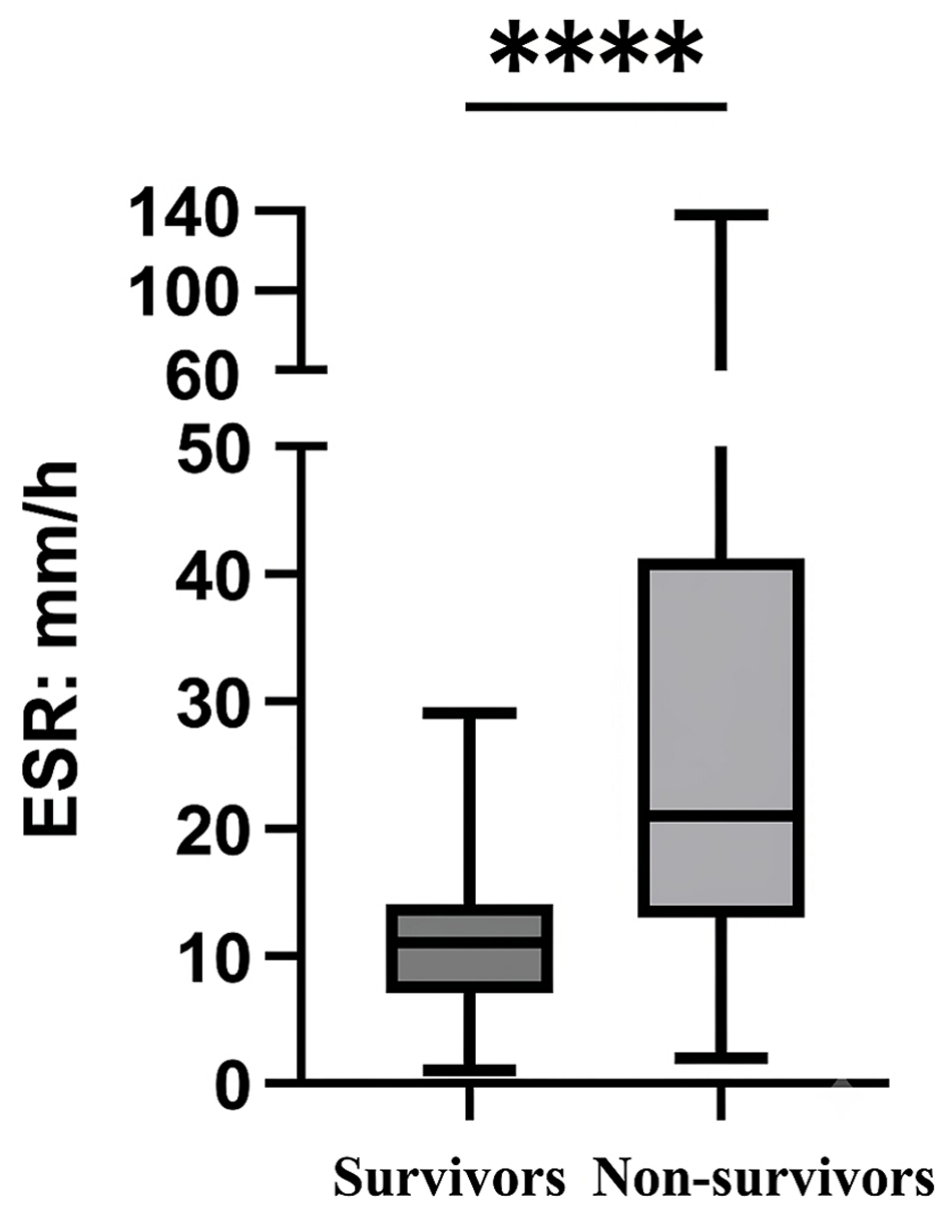
Comparison of ESR levels between survivors and non-survivors in dogs with CKD. Box-and-whisker plots show ESR values in survivors and non-survivors among dogs with CKD. Boxes represent interquartile ranges, horizontal lines represent medians, and whiskers represent ranges. The median ESR value was 11 mm/h in survivors and 21 mm/h in non-survivors. Non-survivors had a significantly higher ESR than survivors. ESR, erythrocyte sedimentation rate; CKD, chronic kidney disease. Significance level is indicated as follows: ****p < 0.0001.

### 3.3 Variables associated with ESR

Correlation analysis was performed to evaluate associations between ESR and clinicopathological variables in dogs with CKD. Significant positive correlations were observed between ESR and serum creatinine (r = 0.36, *p* < 0.0001), phosphate (r = 0.26, *p* = 0.003), potassium (r = 0.20, *p* = 0.029), Ca × P (r = 0.29, *p* = 0.001), MMVD stage C or higher (r = 0.35, *p* < 0.0001), systemic hypertension (r = 0.25, *p* = 0.005), and proteinuria (r = 0.29, *p* = 0.002). In contrast, ESR showed a significant negative correlation with the A/G ratio (r = −0.23, *p* = 0.008). No significant correlations were observed between ESR and the remaining variables listed in Supplementary Table S2.

Multiple linear regression analysis was performed to identify variables associated with ESR in dogs with CKD. Serum creatinine was significantly associated with ESR, with an estimate of 3.3 and a 95% confidence interval of 1.4–6.15 (*p* = 0.002). The Ca × P was also significantly associated with ESR, with an estimate of 1.1 and a 95% confidence interval of 0.31–2.68 (*p* = 0.01).

The A/G ratio was negatively associated with ESR, with an estimate of −25.72 and a 95% confidence interval of −43.09 to −8.34 (*p* = 0.04). Potassium was positively associated with ESR, with an estimate of 4.4 and a 95% confidence interval of 0.76–8.04 (*p* = 0.02). The presence of MMVD stage C or higher was also associated with ESR, with an estimate of 12.92 and a 95% confidence interval of 6.12–19.72 (*p* < 0.001).

Proteinuria and systemic hypertension were not significantly associated with ESR in this model. The estimate was 1.04 for proteinuria (95% confidence interval, −6.9 to 8.98; *p* = 0.79) and 4.5 for systemic hypertension (95% confidence interval, −1.54 to 10.7; *p* = 0.14). The regression results are summarized in Supplementary Table S3.

### 3.4 Variables associated with all-cause mortality

Continuous clinicopathological variables were compared between survivors and non-survivors in the CKD group. No significant differences were observed in body weight, age, hematocrit, reticulocyte percentage, CRP, NLR, PLR, potassium, or bicarbonate.

Non-survivors had a significantly higher ESR than survivors, as described above. The A/G ratio was significantly lower in non-survivors than in survivors (*p* = 0.032). The WBC count was significantly higher in non-survivors than in survivors (*p* = 0.017).

Renal and mineral variables also differed between the two groups. The median BUN concentration was 32 mg/dL in survivors and 60 mg/dL in non-survivors (*p* < 0.0001). The median serum creatinine concentration was 1.2 mg/dL in survivors and 2.6 mg/dL in non-survivors (*p* < 0.0001). The median Ca × P was 45.28 mg^2^/dL^2^ in survivors and 69.41 mg^2^/dL^2^ in non-survivors (*p* < 0.001). These results are summarized in Table 3.

**Table 3:** Comparison of clinicopathological variables between survivors and non-survivors in dogs with CKD.

Variables	Survivors, median (IQR)	Non-survivors, median (IQR)	*p* value
Body weight (kg)	6.8 (4.9–8)	6.5 (4.6–8.3)	0.798
Age (years)	12.2 (9.5–15)	13.5 (11–16)	0.393
HCT (%)	38.4 (32.3–43)	36.5 (30.5–43)	0.584
Reticulocytes (%)	0.8 (0.45–1.4)	0.7 (0.38–1.3)	0.224
ESR (mm/h)	11 (8–14)	21 (13–40)	<0.0001
CRP (mg/dL)	1.8 (0.65–5.9)	2.1 (0.8–7.3)	0.334
A/G (ratio)	0.8 (0.7–0.9)	0.7 (0.6–0.9)	0.032
NLR (ratio)	4.9 (3.6–8.7)	5.25 (4–9.35)	0.793
PLR (ratio)	166.7 (120–288.5)	184 (87.7–330)	0.76
WBC count (10^9^/L)	12.6 (8.8–16.5)	15.3 (9.8–23)	0.017
BUN (mg/dL)	32 (19.5–54)	60 (37.8–88)	<0.0001
CREA (mg/dL)	1.2 (0.8–1.7)	2.6 (1.5–4.5)	<0.0001
CaxP (mg^2^/dL^2^)	45.28 (39.62–54.68)	69.41 (50.63–113.1)	<0.001
Potassium (mmol/L)	4.2 (3.9–4.5)	4.4 (4–4.8)	0.095
HCO3- (mmol/L)	22 (19.4–24.5)	22 (17–23.8)	0.071

Data are presented as median and interquartile range. Continuous clinicopathological variables were compared between survivors and non-survivors in the CKD group using the unpaired *t*-test or Mann–Whitney *U* test according to data distribution. CKD, chronic kidney disease; HCT, hematocrit; ESR, erythrocyte sedimentation rate; CRP, C-reactive protein; A/G, albumin-to-globulin ratio; NLR, neutrophil-to-lymphocyte ratio; PLR, platelet-to-lymphocyte ratio; WBC, white blood cell count; BUN, blood urea nitrogen; CREA, creatinine; Ca×P, calcium-phosphorus product; HCO3−, bicarbonate.

Categorical clinical risk factors were compared between survivors and non-survivors. Stage C or higher MMVD was observed in 9 of 75 survivors (12.0%) and 22 of 52 non-survivors (42.3%; *p* < 0.0001). Proteinuria was observed in 8 of 75 survivors (10.7%) and 18 of 52 non-survivors (34.6%; *p* = 0.002). Systemic hypertension was observed in 23 of 75 survivors (30.7%) and 22 of 52 non-survivors (42.3%), but this difference was not statistically significant (*p* = 0.19). These results are summarized in Table 4.

**Table 4:** Distribution of categorical clinical risk factors according to survival status in dogs with CKD.

Variables	Survivors, *n* (%)	Non-survivors, *n* (%)	*p* value
MMVD stage C or higher	9/75 (12.0%)	22/52 (42.3%)	<0.0001
Systemic hypertension (SBP ≥ 160 mmHg)	23/75 (30.7%)	22/52 (42.3%)	0.19
Proteinuria (UPC > 0.5)	8/75 (10.7%)	18/52 (34.6%)	0.002

Data are presented as number and percentage. Categorical clinical risk factors were compared between survivors and non-survivors using Fisher’s exact test. Percentages were calculated within each survival group. CKD, chronic kidney disease; MMVD, myxomatous mitral valve disease.

Receiver operating characteristic curve analysis was performed to determine cohort-derived cut-off values for variables associated with all-cause mortality in dogs with CKD (Table 5). The ESR had an ROC-AUC of 0.79 (95% CI, 0.71–0.88; *p* < 0.0001); at the optimal cut-off of ≥16 mm/h, the sensitivity and specificity were 64.58% (95% CI, 50.44–76.57) and 81.01% (95% CI, 71.01–88.14), respectively. Among the other variables, serum creatinine (ROC-AUC, 0.78), Ca × *p* (0.77), and blood urea nitrogen (0.72) showed the highest discrimination, whereas the A/G ratio (0.62) and white blood cell count (0.61) showed limited discrimination.

**Table 5:** Cut-off values derived from receiver operating characteristic curve analysis for mortality prediction in dogs with CKD.

Variables	ROC cutoff	Sensitivity (95% CI)	Specificity (95% CI)	ROC-AUC (95% CI)	*p* value
Body weight (kg)	Not estimated (NE)				
Age (years)	NE				0.134
Hematocrit (%)	NE				
Reticulocytes (%)	NE				
ESR (mm/h)	16	64.58 (50.44–76.57)	81.01 (71.01–88.14)	0.79 (0.71–0.88)	<0.0001
CRP (mg/dL)	NE				
A/G (ratio)	0.6	27.08 (16.57–41)	96.2 (89.42–98.86)	0.62 (0.51–0.72)	0.029
NLR (ratio)	NE				
PLR (ratio)	NE				
WBC count (10^9^/L)	18.5	37.5 (25.22–51.64)	84.81 (75.3–91.09)	0.61 (0.51–0.71)	0.036
BUN (mg/dL)	45	70.83 (56.82–81.76)	68.35 (57.45–77.55)	0.72 (0.63–0.82)	<0.0001
CREA (mg/dL)	2.5	54.17 (40.29–67.42)	91.14 (82.82–95.64)	0.78 (0.69–0.86)	<0.0001
CaxP (mg^2^/dL^2^)	50	80.3 (67.54–88.98)	64.94 (53.8–74.65)	0.77 (0.68–0.86)	<0.0001
Potassium (mmol/L)	NE				
HCO_3_^-^ (mmol/L)	NE				

Receiver operating characteristic curve analysis was performed to determine optimal cut-off values for mortality prediction in dogs with CKD. Cut-off values were determined using the Youden index. Sensitivity and specificity are presented as percentages. CKD, chronic kidney disease; ROC, receiver operating characteristic; AUC, area under the curve; CI, confidence interval; ESR, erythrocyte sedimentation rate; CRP, C-reactive protein; A/G, albumin-to-globulin ratio; NLR, neutrophil-to-lymphocyte ratio; PLR, platelet-to-lymphocyte ratio; WBC, white blood cell count; BUN, blood urea nitrogen; CREA, creatinine; Ca×P, calcium-phosphorus product; HCO3−, bicarbonate; NE, not estimated.

Age did not show significant prognostic performance in ROC analysis (*p* = 0.134). Cut-off values were not derived from ROC analysis for body weight, hematocrit, reticulocyte percentage, CRP, NLR, PLR, potassium, or bicarbonate. The results of the ROC analysis are summarized in Table 5.

### 3.5 Cox proportional hazards regression analysis and exploratory composite score construction

Cox proportional hazards regression analysis was performed to evaluate variables associated with all-cause mortality in dogs with CKD. In univariable analysis, ESR ≥ 16 mm/h was associated with mortality, with a hazard ratio of 4.10 and a 95% confidence interval of 2.28–7.68 (*p* < 0.0001).

Other variables associated with mortality in univariable analysis included WBC ≥ 18.5 × 10^9^/L, BUN ≥45 mg/dL, serum creatinine ≥2.5 mg/dL, Ca × *p* ≥ 50 mg^2^/dL^2^, A/G ratio ≤0.6, proteinuria, and MMVD stage C or higher. The hazard ratio was 2.37 for WBC ≥ 18.5 × 10^9^/L (*p* = 0.006), 5.43 for BUN ≥45 mg/dL (*p* < 0.0001), 7.49 for serum creatinine ≥2.5 mg/dL (*p* < 0.0001), 3.18 for Ca × *p* ≥ 50 mg^2^/dL^2^ (*p* < 0.001), 3.00 for A/G ratio ≤0.6 (*p* = 0.002), 3.08 for proteinuria (*p* < 0.001), and 3.49 for MMVD stage C or higher (*p* < 0.0001). Systemic hypertension was not significantly associated with mortality in univariable analysis (*p* = 0.066).

Two multivariable Cox regression models were constructed to examine the prognostic contribution of ESR. In the model incorporating ESR, ESR ≥ 16 mm/h remained associated with all-cause mortality, with a hazard ratio of 3.42 and a 95% confidence interval of 1.78–6.64 (*p* < 0.001). Serum creatinine ≥2.5 mg/dL, BUN ≥45 mg/dL, and MMVD stage C or higher were also associated with mortality. The hazard ratio was 6.37 for BUN ≥45 mg/dL (*p* < 0.0001), 3.19 for serum creatinine ≥2.5 mg/dL (*p* < 0.001), and 2.10 for MMVD stage C or higher (*p* = 0.042).

Proteinuria and systemic hypertension were evaluated as predefined binary clinical variables. In univariable Cox regression analysis, proteinuria was associated with mortality, whereas systemic hypertension was not. Neither proteinuria nor systemic hypertension remained associated with mortality in the multivariable model incorporating ESR.

In the model excluding ESR, BUN ≥45 mg/dL, serum creatinine ≥2.5 mg/dL, and MMVD stage C or higher remained associated with mortality. The hazard ratio was 4.17 for BUN ≥45 mg/dL (*p* < 0.001), 3.31 for serum creatinine ≥2.5 mg/dL (*p* < 0.001), and 2.53 for MMVD stage C or higher (*p* = 0.007). The complete Cox regression results are presented in Table 6.

**Table 6:** Cox proportional hazards regression analysis of ROC-dichotomized and predefined categorical variables in dogs with CKD.

Univariable	Hazard Ratio	95% CI	*p* value
ESR ≥ 16 (mm/h)	4.1	2.28–7.68	<0.0001
WBC count ≥ 18.5 (10^9^/L)	2.37	1.29–4.21	0.006
BUN ≥ 45 (mg/dL)	5.43	2.91–10.69	<0.0001
CREA ≥ 2.5 (mg/dL)	7.49	4.15–13.68	<0.0001
CaxP ≥ 50 (mg^2^/dL^2^)	3.18	1.70–6.38	<0.001
A/G ≤ 0.6 (ratio)	3.0	1.56–5.49	0.002
Proteinuria (UPC > 0.5)	3.08	1.68–5.48	<0.001
MMVD Stage C or higher	3.49	1.96–6.17	<0.0001
Systemic hypertension (SBP ≥ 160 mmHg)	1.68	0.97–2.92	0.066
Multivariable (without ESR)	Hazard Ratio	95% CI	*p* value
WBC count ≥ 18.5 (10^9^/L)	1.21	0.56–2.51	0.619
BUN ≥ 45 (mg/dL)	4.17	1.86–9.6	<0.001
CREA ≥ 2.5 (mg/dL)	3.31	1.74–6.56	<0.001
CaxP ≥ 50 (mg^2^/dL^2^)	0.69	0.34–1.43	0.31
A/G ≤ 0.6 (ratio)	1.14	0.47–2.71	0.77
Proteinuria (UPC > 0.5)	1.36	0.72–2.51	0.34
MMVD Stage C or higher	2.53	1.3–4.83	0.007
Systemic hypertension (SBP ≥ 160 mmHg)	1.2	0.62–2.25	0.59
Multivariable (with ESR)	Hazard Ratio	95% CI	*p* value
ESR ≥ 16 (mm/h)	3.42	1.78–6.64	<0.001
WBC count ≥ 18.5 (10^9^/L)	1.08	0.48–2.31	0.85
BUN ≥ 45 (mg/dL)	6.37	2.64–16.04	<0.0001
CREA ≥ 2.5 (mg/dL)	3.19	1.69–6.2	<0.001
CaxP ≥ 50 (mg^2^/dL^2^)	0.8	0.4–1.65	0.54
A/G ≤ 0.6 (ratio)	1.14	0.49–2.56	0.75
Proteinuria (UPC > 0.5)	0.9	0.46–1.73	0.76
MMVD Stage C or higher	2.1	1.03–4.19	0.042
Systemic hypertension (SBP ≥ 160 mmHg)	1.01	0.51–1.97	0.97

Univariable and multivariable Cox proportional hazards regression analyses were performed to evaluate variables associated with mortality in dogs with CKD. Continuous variables were dichotomized according to ROC-derived cut-off values. Proteinuria, systemic hypertension, and MMVD stage C or higher were entered as predefined binary categorical variables. Proteinuria was defined as UPC >0.5, systemic hypertension was defined as SBP ≥160 mmHg, and MMVD stage C or higher was defined according to established veterinary cardiology guidelines. Each categorical variable was coded as 1 when present and 0 when absent. Two multivariable models were constructed: one excluding ESR and one incorporating ESR. Hazard ratios greater than 1 indicate increased risk of mortality. CKD, chronic kidney disease; ESR, erythrocyte sedimentation rate; HR, hazard ratio; CI, confidence interval; WBC, white blood cell count; BUN, blood urea nitrogen; CREA, creatinine; Ca×P, calcium-phosphorus product; A/G, albumin-to-globulin ratio; UPC, urine protein-to-creatinine ratio; SBP, systolic blood pressure; MMVD, myxomatous mitral valve disease.

Exploratory composite risk scores were constructed using beta coefficients derived from the multivariable Cox regression models. The score derived from the model without ESR was defined as the multi-biomarker CKD risk score without ESR, or MCB score. The score derived from the model incorporating ESR was defined as the multi-biomarker CKD risk score incorporating ESR, or MCBE score.

The MCBE score included MMVD stage C or higher, serum creatinine ≥2.5 mg/dL, BUN ≥45 mg/dL, and ESR ≥ 16 mm/h. Based on beta coefficient-derived weighting, 2 points were assigned to MMVD stage C or higher, 3 points to serum creatinine ≥2.5 mg/dL, 5 points to BUN ≥45 mg/dL, and 3 points to ESR ≥ 16 mm/h. The MCBE score was calculated as follows:

MCBE score = 2 × (MMVD stage C or higher) + 3 × (serum creatinine ≥2.5 mg/dL) + 5 × (BUN ≥45 mg/dL) + 3 × (ESR ≥ 16 mm/h).

The MCB score included MMVD stage C or higher, serum creatinine ≥2.5 mg/dL, and BUN ≥45 mg/dL. Based on beta coefficient-derived weighting, 2 points were assigned to MMVD stage C or higher, 2.5 points to serum creatinine ≥2.5 mg/dL, and 3 points to BUN ≥45 mg/dL. The MCB score was calculated as follows:

MCB score = 2 × (MMVD stage C or higher) + 2.5 × (serum creatinine ≥2.5 mg/dL) + 3 × (BUN ≥45 mg/dL).

The beta coefficients, coefficient ratios, assigned weights, and score equations are summarized in Table 7.

**Table 7:** Multi-biomarker risk scores based on beta coefficients derived from Cox proportional hazards regression analysis.

Variables	Beta coefficient	Ratio	Assigned points	MCBE score
MMVD stage C or higher	0.74	1	2	2*(MMVD Stage C ≥) + 3*(CREA ≥ 2.5) + 5*(BUN ≥ 45) + 3*(ESR ≥ 16)
CREA ≥ 2.5 (mg/dL)	1.16	1.57	3	
BUN ≥ 45 (mg/dL)	1.85	2.5	5	
ESR ≥ 16 (mm/h)	1.23	1.66	3	

Variables	Beta coefficient	Ratio	Assigned points	MCB score
MMVD stage C or higher	0.93	1	2	2*(MMVD Stage C ≥) + 2.5*(CREA ≥ 2.5) + 3*(BUN ≥ 45)
CREA ≥ 2.5 (mg/dL)	1.2	1.29	2.5	
BUN ≥ 45 (mg/dL)	1.43	1.53	3	

Multi-biomarker risk scores were constructed using beta coefficients derived from the multivariable Cox proportional hazards regression models. The MCBE score was derived from the model incorporating ESR, whereas the MCB score was derived from the model excluding ESR. Coefficient ratios were calculated relative to the smallest beta coefficient in each model, and assigned weights were rounded for clinical applicability. Each risk factor was coded as 1 when present and 0 when absent. ESR, erythrocyte sedimentation rate; MCB, multi-biomarker CKD risk score without ESR; MCBE, multi-biomarker CKD risk score incorporating ESR; CKD, chronic kidney disease; MMVD, myxomatous mitral valve disease; CREA, creatinine; BUN, blood urea nitrogen.

### 3.6 Apparent prognostic performance and survival stratification of ESR, MCB score, and MCBE score

Receiver operating characteristic curve analysis was performed to describe the apparent prognostic performance of ESR, MCB score, and MCBE score for all-cause mortality prediction in dogs with CKD. When used alone, ESR had an ROC-AUC of 0.79, with a 95% confidence interval of 0.71–0.88 (*p* < 0.0001). At a cut-off value of ≥16 mm/h, the sensitivity was 64.58%, and the specificity was 81.01%.

The MCB score had an ROC-AUC of 0.85, with a 95% confidence interval of 0.79–0.92 (*p* < 0.0001). At a cut-off value of ≥2, the sensitivity was 96.15%, and the specificity was 65.33%.

The MCBE score had an ROC-AUC of 0.87, with a 95% confidence interval of 0.81–0.93 (*p* < 0.0001). At a cut-off value of ≥6, the sensitivity was 71.15%, and the specificity was 86.67%.

The ROC-AUC of the MCBE score was numerically higher than that of the MCB score, but the difference was not statistically significant (paired DeLong test, *p* = 0.39). The MCBE score showed higher specificity, whereas the MCB score showed higher sensitivity. These ROC analysis results are summarized in Table 8.

**Table 8:** Receiver operating characteristic curve analysis of ESR, MCB score, and MCBE score in dogs with CKD.

Variables	ROC-AUC (95% CI)	Cut-off	Sensitivity, % (95% CI)	Specificity, % (95% CI)
ESR	0.79 (0.71–0.88)	≥16 mm/h	64.58 (50.44–76.57)	81.01 (71.01–88.14)
MCB score	0.85 (0.79–0.92)	≥2	96.15 (87.02–99.32)	65.33 (54.05–75.12)
MCBE score	0.87 (0.81–0.93)	≥6	71.15 (57.73–81.67)	86.67 (77.17–92.59)

Receiver operating characteristic curve analysis was performed to compare the prognostic performance of ESR, MCB score, and MCBE score for mortality prediction in dogs with CKD. Cut-off values were determined using the Youden index. Sensitivity and specificity are presented as percentages. CKD, chronic kidney disease; ROC, receiver operating characteristic; AUC, area under the curve; CI, confidence interval; ESR, erythrocyte sedimentation rate; MCB, multi-biomarker CKD risk score without ESR; MCBE, multi-biomarker CKD risk score incorporating ESR. The ROC-AUCs of the MCB and MCBE scores were compared using the paired DeLong test (*p* = 0.39).

Kaplan–Meier survival analysis was performed after stratifying dogs with CKD into four ordered groups according to ESR, MCB score, and MCBE score. The labels Q1 to Q4 indicate progressively higher ESR values or progressively higher composite risk scores. The maximum follow-up duration was 786 days.

In the ESR-based analysis, median survival time was not reached in Q1 and Q2. Median survival time was 245 days in Q3 and 35 days in Q4.

In the MCB score-based analysis, median survival time was not reached in Q1. Median survival time was 308 days in Q2, 275 days in Q3, and 50 days in Q4.

In the MCBE score-based analysis, median survival time was not reached in Q1 and Q2. Median survival time was 200 days in Q3 and 31 days in Q4. Higher MCBE score groups showed shorter median survival times in this cohort.

Median survival times stratified by ESR, MCB score, and MCBE score groups are summarized in Supplementary Table S4. Kaplan–Meier survival curves are presented in Figure 3.

**Figure 3:**
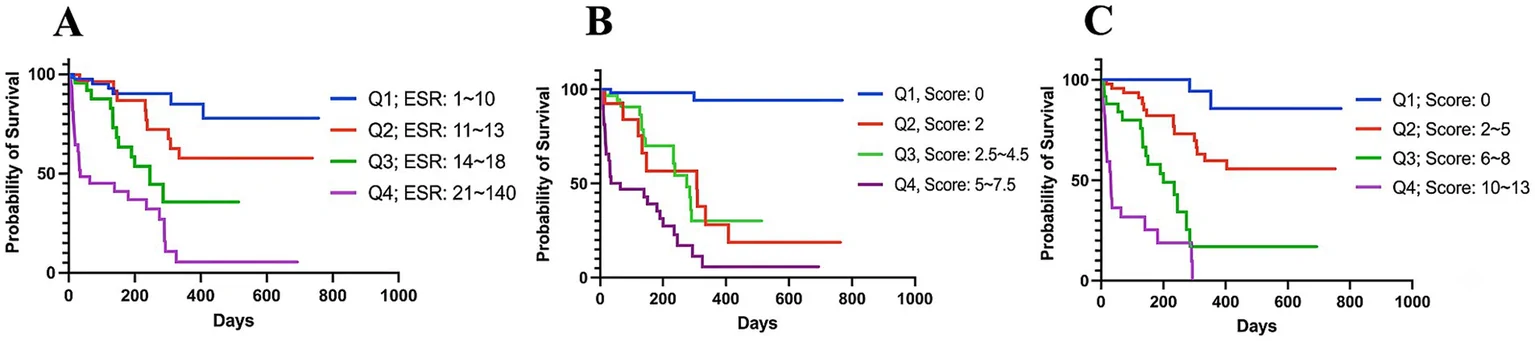
Kaplan–Meier survival analysis according to ESR, MCB score, and MCBE score. Kaplan–Meier survival curves were generated after dogs with CKD were stratified into quartiles according to ESR, MCB score, and MCBE score. Panel **(A)** shows survival curves according to ESR quartiles: Q1, 1–10 mm/h; Q2, 11–13 mm/h; Q3, 14–18 mm/h; and Q4, 21–140 mm/h. Panel **(B)** shows survival curves according to MCB score quartiles: Q1, score 0; Q2, score 2; Q3, score 2.5–4.5; and Q4, score 5–7.5. Panel **(C)** shows survival curves according to MCBE score quartiles: Q1, score 0; Q2, score 2–5; Q3, score 6–8; and Q4, score 10–13. Median survival times for each quartile are summarized in Supplementary Table S4. ESR, erythrocyte sedimentation rate; CKD, chronic kidney disease; MCB, multi-biomarker CKD risk score without ESR; MCBE, multi-biomarker CKD risk score incorporating ESR. Survival curves were compared using the log-rank test.

## 4 Discussion

The present study evaluated the clinical and prognostic relevance of ESR in dogs with CKD and constructed an exploratory multi-biomarker CKD risk score incorporating ESR. The ESR was significantly higher in dogs with CKD than in healthy control dogs, particularly in dogs with advanced IRIS stages. Moreover, ESR was also significantly higher in non-survivors than in survivors and remained associated with all-cause mortality in multivariable Cox regression analysis. In addition, the ESR-inclusive MCBE score showed a numerically higher apparent ROC-AUC and higher specificity than the MCB score in this cohort, although the difference in ROC-AUC was not statistically significant. These findings suggest that ESR may provide complementary prognostic information in dogs with CKD, although the score should be interpreted as exploratory and not as a validated clinical prediction model.

The ESR is a nonspecific hematological marker affected by erythrocyte aggregation, plasma protein composition, erythrocyte mass, and inflammatory status. Fibrinogen and immunoglobulins reduce the negative surface charge of erythrocytes and promote rouleaux formation, thereby increasing sedimentation velocity (1, 2). In dogs, ESR has been reported to differ between healthy and diseased populations and to show weak-to-moderate associations with inflammatory biomarkers, including CRP, NLR, fibrinogen, and A/G ratio (2, 3). A recent canine study also suggested that ESR may have prognostic value in inflammatory diseases (4). Therefore, the present findings are consistent with previous veterinary studies indicating that ESR can reflect systemic disease activity, although it is not disease-specific (1–4).

In this study, ESR was higher in dogs with CKD than in healthy control dogs. However, ESR did not differ significantly between healthy controls and dogs with CKD stage 1 or stage 2, whereas dogs with CKD stage 3 and stage 4 showed significantly higher ESR values. This pattern may reflect the limited systemic impact of early CKD. Although dogs with CKD stage 1 and stage 2 were older than healthy control dogs descriptively, their ESR values were not significantly different from those of healthy controls. In addition, dogs with CKD stage 4 had the highest ESR values despite having a lower median age than dogs with CKD stage 3. These findings suggest that ESR elevation was not explained by age distribution alone.

In stage 1 and stage 2 CKD, renal excretory function may be preserved or only mildly impaired, and early reductions in renal function or mild structural renal changes may not be sufficient to produce measurable changes in ESR. In contrast, as CKD progresses, dogs may develop uremia, anemia, mineral imbalance, metabolic acidosis, proteinuria, systemic hypertension, nutritional deterioration, and cardiovascular comorbidity (5, 6). These systemic complications are more prominent in advanced CKD and may influence ESR through chronic inflammation, altered plasma protein composition, and cumulative disease burden. Therefore, the significant ESR elevation observed in CKD stage 3 and stage 4 may reflect advanced systemic consequences of CKD rather than renal excretory dysfunction alone.

The prognostic value of ESR was supported by its higher values in non-survivors and by the ROC-derived cut-off value of 16 mm/h. In human medicine, ESR is frequently elevated in chronic renal failure and end-stage renal disease, although it is influenced by anemia, plasma proteins, and non-renal factors (17–19). In dogs, survival in CKD has been associated with multiple variables, including creatinine, BUN, phosphorus, calcium-phosphorus product, proteinuria, body condition, and hematological abnormalities (7–10, 12). These data support the concept that prognosis in canine CKD is not determined by a single renal marker. The ESR may contribute additional information by reflecting chronic inflammatory, protein-related, and comorbid disease burden.

This interpretation is consistent with the concept that CKD is a systemic syndrome rather than a disorder limited to reduced glomerular filtration. In CKD, uremic toxin accumulation, oxidative stress, chronic inflammation, altered plasma protein composition, and cardiovascular comorbidity may develop in parallel. Therefore, the nonspecific nature of ESR may be clinically relevant in this setting, because ESR can integrate several downstream consequences of CKD that are not fully captured by static renal excretory markers.

Correlation and multiple linear regression analyses showed that ESR was associated with creatinine, Ca × P, A/G ratio, potassium, and MMVD stage C or higher. These associations are biologically plausible but should be interpreted cautiously. Creatinine reflects renal excretory function, whereas Ca × P reflects mineral metabolism; both have been associated with CKD severity or survival in previous canine studies (5, 9, 10). The association between ESR and Ca × P may also indicate that ESR increases in dogs with advanced mineral-metabolic derangement; however, because markers of CKD-mineral and bone disorder or vascular calcification were not evaluated, ESR should not be interpreted as a direct marker of mineralization. Furthermore, CKD is also associated with chronic inflammation, oxidative stress, uremic toxin accumulation, and cardiovascular complications, which may contribute to systemic disease burden (20–22). The absence of significant ESR elevation in stage 1 and stage 2 CKD suggests that mild renal dysfunction or early structural renal disease alone may not substantially affect ESR. In contrast, the association of ESR with creatinine, Ca × P, A/G ratio, and MMVD stage C or higher suggests that ESR becomes more clinically informative when renal dysfunction is accompanied by metabolic, protein-related, inflammatory, or cardiovascular abnormalities. However, these mechanisms were not directly measured in this study. Therefore, the observed associations should be interpreted as indirect evidence that ESR is related to multiple clinical dimensions of CKD.

The negative association between ESR and A/G ratio is consistent with the known influence of plasma proteins on erythrocyte sedimentation. In CKD, chronic inflammation and uremia-associated oxidative stress may alter the acute-phase protein profile, including increased globulin and fibrinogen concentrations (20–22). These proteins reduce erythrocyte zeta potential and promote rouleaux formation, thereby increasing ESR. In contrast, albumin may decrease in chronic inflammatory, protein-losing, or malnourished states. Therefore, a reduced A/G ratio may represent a protein-related inflammatory milieu that facilitates ESR elevation in dogs with CKD. Because fibrinogen, immunoglobulin fractions, and other acute-phase proteins were not measured, their direct contribution to ESR elevation could not be confirmed in this study.

The presence of MMVD stage C or higher was associated with both ESR and mortality. This finding is clinically relevant because cardiovascular and renal disorders frequently interact in dogs. The cardiovascular-renal axis disorder concept describes bidirectional interactions between cardiac and renal dysfunction mediated by hemodynamic, neurohormonal, inflammatory, and metabolic pathways (13, 23). In dogs with chronic mitral valve disease, renal dysfunction has been associated with more advanced cardiac disease and shorter survival (14). The present findings do not indicate that ESR directly measures cardiovascular-renal interaction, but they suggest that ESR may increase in CKD dogs with greater comorbid disease burden (13–15, 23).

In multivariable Cox regression analysis, ESR ≥ 16 mm/h remained associated with mortality after adjustment for BUN, creatinine, and MMVD stage C or higher. Creatinine and BUN are conventional markers of renal dysfunction and were strong prognostic variables in this cohort (5–7). However, ESR may provide additional prognostic information because it reflects biological processes not fully captured by renal excretory markers, including altered plasma protein composition, chronic inflammatory activity, and comorbidity (1, 17–22, 24). However, residual confounding cannot be excluded because treatment differences, nutritional status, occult inflammatory disease, and unmeasured inflammatory proteins may have influenced both ESR and survival.

The MCBE score was constructed to integrate ESR with conventional prognostic variables. In this cohort, the MCBE score had a numerically higher ROC-AUC than the MCB score, although the difference was not statistically significant (*p* = 0.39), and it showed higher specificity but lower sensitivity. In Kaplan–Meier analysis, the MCB score showed less consistent intermediate-risk stratification, with median survival times of 308 days in Q2 and 275 days in Q3. In contrast, the MCBE score showed more orderly separation of survival curves, with unreached median survival in Q1 and Q2 and shorter median survival times in Q3 and Q4. These findings suggest that ESR may help bridge the gap between conventional renal or cardiovascular variables and the actual systemic deterioration of the patient. However, the MCB and MCBE scores were derived and evaluated in the same cohort. Therefore, the observed performance represents apparent prognostic performance and should not be interpreted as validated predictive superiority. In addition, because the MCBE score contained all components of the MCB score and this study was not powered to detect small differences in ROC-AUC, the non-significant difference between the two scores does not exclude a prognostic contribution of ESR, which remained independently associated with mortality in the multivariable Cox model; this should be confirmed in an independent cohort.

This study has several limitations. First, this was a single-center retrospective study; therefore, the findings may not be generalizable to all canine CKD populations, and data collection and follow-up were not standardized. Second, healthy control dogs and CKD dogs were not age-matched. Because ESR can be affected by age and chronic systemic conditions, comparisons between healthy controls and CKD dogs may have been influenced by age-related confounding. However, within the CKD cohort, age was not significantly correlated with ESR, and age did not show significant prognostic performance in ROC analysis. In addition, age did not differ significantly between survivors and non-survivors, whereas ESR was markedly higher in non-survivors. Therefore, age imbalance should be considered when interpreting comparisons between healthy controls and CKD dogs, but it is unlikely to fully explain the association between ESR and mortality observed within the CKD group. Third, the number of dogs with IRIS stage 3 and stage 4 CKD was relatively small, which may affect stage-specific comparisons. Fourth, survival analysis was based on all-cause mortality rather than renal-specific mortality. Because dogs with CKD are often geriatric and may have concurrent diseases, death may not always have been directly attributable to CKD.

Fifth, ESR values were not corrected for hematocrit, and hematocrit, total protein, and globulin concentrations were not included as covariates in the regression or Cox models because they were not significantly correlated with ESR in this cohort. Because anemia becomes more frequent and severe as CKD progresses, and a lower hematocrit accelerates erythrocyte sedimentation, part of the ESR elevation in advanced CKD may reflect anemia and altered plasma protein composition rather than inflammatory activity alone; therefore, residual confounding by these factors cannot be excluded. Additional limitations include non-standardized treatment, baseline-only ESR measurement, and the absence of direct measurements of fibrinogen, immunoglobulin fractions, serum amyloid A, cytokines, iron status, or erythropoietic markers. Potential collinearity among CKD severity-related variables, including BUN, creatinine, and Ca × P, may also have influenced the multivariable Cox estimates and the assigned weights of the composite scores. Finally, the MCBE score requires external validation, preferably in a prospective multicenter cohort, before clinical use.

## 5 Conclusion

Baseline ESR was associated with CKD severity and all-cause mortality in dogs with CKD. Baseline ESR was significantly higher in dogs with advanced IRIS CKD stages and in non-survivors, and an ESR cut-off value of 16 mm/h remained associated with mortality in multivariable Cox regression analysis.

Incorporation of ESR into a multi-biomarker CKD risk score yielded a numerically higher, but not significantly different, apparent ROC-AUC and more orderly survival stratification than the score excluding ESR in this cohort. These findings support ESR as a complementary prognostic biomarker in canine CKD. However, because the MCBE score was developed and evaluated in the same cohort, prospective external validation is required before clinical implementation.

## Data Availability

The data that support the findings of this study are available from the corresponding author upon reasonable request.

## Glossary

A/G: albumin-globulin ratio
AKI: acute kidney injury
AUC: area under the curve
BCS: body condition score
BUN: blood urea nitrogen
Ca × P: calcium-phosphorus product
CBC: complete blood count
CKD: chronic kidney disease
CREA: creatinine
CRP: C-reactive protein
CvRD: cardiovascular-renal axis disorder
ESR: erythrocyte sedimentation rate
GFR: glomerular filtration rate
HCO_3_: bicarbonate
HCT: hematocrit
HR: hazard ratio
IACUC: Institutional Animal Care and Use Committee
IRIS: International Renal Interest Society
K: potassium
K3-EDTA: tripotassium ethylenediaminetetraacetic acid
MCB: multi-biomarker CKD risk score without ESR
MCBE: multi-biomarker CKD risk score incorporating ESR
MMVD: myxomatous mitral valve disease
MST: median survival time
NLR: neutrophil-lymphocyte ratio
OS: overall survival
PLR: platelet-lymphocyte ratio
ROC: receiver operating characteristic curve
SBP: systolic blood pressure
SDMA: symmetric dimethylarginine
UPC: urine protein-to-creatinine ratio
USG: urine specific gravity
WBC: white blood cell count
