# Supplementary material for: Baseline erythrocyte sedimentation rate is associated with mortality in dogs with chronic kidney disease
Source: Front Vet Sci. 2026 Oct 22;13:1924110. doi: 10.3389/fvets.2026.1924110 (PMC13735095; doi:10.3389/fvets.2026.1924110)
Supplement: Supplementary file 1 [file Table_1.DOCX]

**Supplementary Table S1.** Comparison of inflammatory biomarkers between healthy control dogs and dogs with CKD.

|  | **Variables** | **ESR (mm/h)** | **CRP (mg/dL)** | **A/G (ratio)** | **NLR (ratio)** | **PLR**  **(ratio)** | **WBC (10^9^/L)** |
| --- | --- | --- | --- | --- | --- | --- | --- |
| **Median (IQR)** | Control  (n = 145) | 9  (3–11) | 0.6  (0.4–1.3) | 0.9  (0.8–1.1) | 2.8  (1.7–4) | 110.3  (80.6–164.4) | 10.3  (7.5–13.1) |
|  | CKD  (n = 127) | 13  (10–18) | 2.1  (0.7–7.2) | 0.8  (0.7–0.9) | 5.1  (3.5–8.8) | 172.4  (110–304) | 13.53  (8.99–18) |
|  | *p value* | <0.0001 | <0.0001 | <0.001 | <0.0001 | <0.0001 | <0.0001 |

Data are presented as median and interquartile range. Inflammatory biomarkers were compared between healthy control dogs and dogs with chronic kidney disease. Statistical comparisons were performed using the unpaired t-test or Mann–Whitney U test according to data distribution. CKD, chronic kidney disease; ESR, erythrocyte sedimentation rate; CRP, C-reactive protein; A/G, albumin-to-globulin ratio; NLR, neutrophil-to-lymphocyte ratio; PLR, platelet-to-lymphocyte ratio; WBC, white blood cell count; IQR, interquartile range.

**Supplementary Table S2.** Correlation analysis between ESR and clinicopathological variables in dogs with CKD.

| **Variables** | **Correlation coefficient (r)** | ***p* value** |
| --- | --- | --- |
| Age | -0.02 | 0.8 |
| Hematocrit | -0.11 | 0.207 |
| Red cell distribution width (RDW) | 0.1 | 0.228 |
| Reticulocytes count | -0.06 | 0.493 |
| White blood cell count | 0.06 | 0.475 |
| Neutrophil count | -0.01 | 0.9 |
| Lymphocyte count | -0.01 | 0.85 |
| Platelet | 0.03 | 0.71 |
| Neutrophil-to-lymphocyte ratio | 0.002 | 0.974 |
| Platelet-to-lymphocyte ratio | 0.04 | 0.627 |
| C-reactive protein | 0.06 | 0.497 |
| Blood urea nitrogen | 0.04 | 0.6 |
| Creatinine | 0.36 | <0.0001 |
| Phosphate | 0.26 | 0.003 |
| Total calcium | -0.02 | 0.755 |
| Cholesterol | 0.19 | 0.1 |
| Total protein | -0.004 | 0.96 |
| Albumin | -0.22 | 0.115 |
| Globulin | 0.12 | 0.17 |
| Potassium | 0.2 | 0.029 |
| Bicarbonate (HCO3-) | -0.18 | 0.068 |
| Albumin-to-globulin ratio | -0.23 | 0.008 |
| CaxP | 0.29 | 0.001 |
| MMVD stage C or higher | 0.35 | <0.0001 |
| Systemic hypertension (SBP ≥160 mmHg) | 0.25 | 0.005 |
| Proteinuria (UPC > 0.5) | 0.29 | 0.002 |

Pearson’s correlation analysis was performed to evaluate associations between ESR and clinicopathological variables in dogs with CKD. Correlation coefficients are presented as r values. Positive r values indicate positive correlations with ESR, whereas negative r values indicate inverse correlations. Categorical variables, including MMVD stage C or higher, hypertension, and proteinuria, were coded as binary variables. CKD, chronic kidney disease; ESR, erythrocyte sedimentation rate; RDW, red cell distribution width; NLR, neutrophil-to-lymphocyte ratio; PLR, platelet-to-lymphocyte ratio; CRP, C-reactive protein; BUN, blood urea nitrogen; HCO3−, bicarbonate; A/G, albumin-to-globulin ratio; Ca×P, calcium-phosphorus product; MMVD, myxomatous mitral valve disease.

**Supplementary Table S3.** Multiple linear regression analysis of variables associated with ESR in dogs with CKD.

| **Variables** | **Estimate** | **95% CI** | ***p* value** |
| --- | --- | --- | --- |
| Creatinine | 3.3 | 1.4-6.15 | 0.002 |
| CaxP | 1.1 | 0.31-2.68 | 0.01 |
| A/G | -25.72 | -43.09 to -8.34 | 0.04 |
| Potassium | 4.4 | 0.76-8.04 | 0.02 |
| MMVD stage C or higher | 12.92 | 6.12-19.72 | <0.001 |
| Proteinuria (UPC > 0.5) | 1.04 | -6.9 to 8.98 | 0.79 |
| Systemic hypertension (SBP ≥160 mmHg) | 4.5 | -1.54 to 10.7 | 0.14 |

Multiple linear regression analysis was performed to identify variables independently associated with ESR in dogs with CKD. Estimates represent the expected change in ESR associated with each variable. Proteinuria, hypertension, and MMVD stage C or higher were coded as binary variables. CKD, chronic kidney disease; ESR, erythrocyte sedimentation rate; CI, confidence interval; Ca×P, calcium-phosphorus product; A/G, albumin-to-globulin ratio; MMVD, myxomatous mitral valve disease.

**Supplementary Table S4.** Median survival times stratified by quartiles of ESR and multi-biomarker risk scores in dogs with CKD.

| **ﾠMedian survival time (95% CI)  [survivors/non-survivors]** | | | |
| --- | --- | --- | --- |
| **Quartile/Variables** | **ESR** | **MCB score** | **MCBE score** |
| **Q1** | Not reached [37/6] | Not reached [49/2] | Not reached [32/2] |
| **Q2** | Not reached [19/9] | 308(73-408) [4/9] | Not reached [31/15] |
| **Q3** | 245(133-infinity) [13/12] | 275(144-infinity) [16/15] | 200(132-275) [9/16] |
| **Q4** | 35(17-235) [6/25] | 50(17-190) [6/26] | 31(15-139) [3/19] |

Dogs with CKD were stratified into quartiles according to ESR, MCB score, and MCBE score. Data are presented as median survival time with 95% confidence interval and the number of survivors and non-survivors in each quartile. Not reached indicates that the median survival time was not reached during the follow-up period. CKD, chronic kidney disease; ESR, erythrocyte sedimentation rate; MCB, multi-biomarker CKD risk score without ESR; MCBE, multi-biomarker CKD risk score incorporating ESR; CI, confidence interval.
